# Polysomnographic survey of sleep architecture in patients with methamphetamine dependence during remission

**DOI:** 10.5935/1984-0063.20200120

**Published:** 2021

**Authors:** Amir Rezaei-Ardani, Fariborz Rezaei-Talab, Lahya Afshari-Saleh, Hadi Asad-Pour, Zahra Amjadi-Goojgi

**Affiliations:** 1 Mashhad University of Medical Sciences, Psychiatry - Mashhad - Khorasan Razavi - Iran.; 2 Mashhad University of Medical Sciences, Neurology - Mashhad - Khorasan Razavi - Iran.; 3 Mashhad University of Medical Sciences, Occupational Medicine - Mashhad - Khorasan Razavi - Iran.

**Keywords:** Sleep, Sleep stages, Methamphetamine, Polysomnography

## Abstract

**Introduction:**

Methamphetamine dependence is common in the world. Methamphetamine affects sleep architecture through changes in the monoaminergic activity of the brain. Limited studies investigated the sleep architecture in patients with methamphetamine dependence during prolonged abstinence. Therefore, this study investigated the sleep architecture of methamphetamine ex-users in the remission phase by polysomnography.

**Material and Methods:**

This was a cross-sectional study conducted during 2015-2017 in Mashhad, Iran. 12 methamphetamine ex-users in early full remission phase were selected from residential treatment centers through the convenient sampling method. The clinical interview was made to confirm the diagnosis and assess the inclusion and exclusion criteria. We performed urine dipstick tests to detect any relapses. Participants underwent a one-night polysomnographic evaluation, voluntarily. The collected data were analyzed by independent sample t-test and chi-square test, using SPSS-16. The level of significance was less than .05.

**Results:**

The mean total sleep time of participants was significantly lower than the total sleep period (333.6±79.1 vs. 403.0±52.9 minutes, respectively; p=0.001), leading to a significant low sleep efficiency (75.7±14.4%, p=0.047). Evaluation of rapid eye movement (REM) sleep showed a significant increase in the REM latency compared to the healthy population (p<0.001). Stages 1 and 3 of non-REM sleep were increased compared to the healthy population, too (p<0.001 and p=0.002, respectively).

**Conclusion:**

Former methamphetamine users continue to experience some long-term abnormalities in sleep architecture a few months after drug cessation

## INTRODUCTION

Sleep disturbances are a group of disorders with a broad impact on people’s health status, quality of life, and productivity^1, 2^. Every year, they impose high costs on the health care system^3^. There are several causes of sleep disturbances, including misuse of amphetamine derivatives^4^.

The main effect of amphetamines on the brain is increasing attention and reducing fatigue and sleep^5^. Also, they enhance the subjective sense of pleasure^6^. Therefore, many of them are sold in illegal markets for recreational use^5^. Amphetamines are well-known stimulants with both medical and illegal uses^7, 8^. Amphetamine-like substances, such as methamphetamine (MA), are the most widely used synthetic drugs worldwide^9^.

Amphetamine derivatives could affect sleep architecture through enhancing catecholamine activity in the brain^10^. Limited studies investigated the sleep architecture in patients with amphetamine dependence during abstinence^11^. Older studies have shown that low doses of MA immediately decrease the amount of sleep, increase sleep onset latency, reduce rapid eye movement (REM) sleep, and induce insomnia^12^, while MA discontinuation often makes drowsiness and an increase in REM sleep for a few nights^13^. Surprisingly, after the first 3-5 days of abstinence, long-term MA abusers experience delayed onset insomnia^13^, which may continue for at least 14 days^14^. Delayed onset insomnia raises questions about MA-induced persisting alteration of CNS sleep-wake mechanisms. However, studies on the effects of amphetamines on sleep architecture usually have some limitations. Many of them are focused on amphetamine-type medications (e.g., methylphenidate), low doses, or single doses of illegal amphetamines. Nonetheless, the residual effects of repeated, high doses of methamphetamines on sleep architecture, as an addictive drug, could be different from prescribed, low doses, or single doses of amphetamines^15^. The half-life of smoked methamphetamine is 8-17 hours, which is much higher than other stimulants^16^. Methamphetamine has documented neurotoxic effects. It contributes to neuronal damage, especially in synaptic terminals, through generating oxidative stress, mitochondrial dysfunction, disruption of the blood-brain barrier, and hyperthermia. It also changes the concentration of neurotransmitters, notably glutamate and dopamine in the synaptic clefts^17, 18^. Despite the current concerns, to our best knowledge, no study has assessed the sleep architecture of patients with MA dependence after long periods of abstinence.

Therefore, this study aimed to paraclinically investigate the sleep architecture of the participants with a diagnosis of MA dependence in the remission phase (at least one month after discontinuation of MA) by polysomnography (PSG).

## MATERIAL AND METHODS

We conducted the present cross-sectional study in 2015-2017 in Mashhad, the second-largest city of Iran. According to the addiction treatment policies of Iran, residential treatment facilities are available for volunteer patients with substance use disorders. Patients are admitted for relatively long-terms (usually 90 days or more) with no access to drugs during the inpatient treatment period. These centers offer a variety of services, including individual and group psychotherapy and over the counter (OTC) medications. Therefore, patients with serious mood or anxiety symptoms, thoughts of self-harm or injury to others, psychosis, or in the intoxication phase are not accepted in these centers. Methamphetamine use disorder without other comorbid psychiatric or medical conditions does not require any specialist intervention^19^; so, residential centers help these patients against craving in their early abstinence phase.

We selected 12 participants through a convenient sampling method from a group of MA-abusers in the residential treatment centers in Mashhad. We explained the study procedure for almost 100 patients. Among them, 41 patients voluntarily agreed to participate in the study. We performed a clinical interview with them and reviewed their medical record to investigate the inclusion and exclusion criteria of the study. At the end of our assessment, 12 patients met the inclusion criteria of the study.

Patients were selected if they were 18-50 years old, fulfilled written informed consent, were diagnosed with MA dependence based on Diagnostic and Statistical Manual of Mental Disorders - Fourth Edition - Text Revision (DSM-IV-TR), had no comorbid use disorder to other substances (except occasional use of alcohol, opiates, benzodiazepines, nicotine, and cannabis), did not use medications that affect sleep cycles during admission in residential centers (e.g., antihistamines), did not have other MA-induced disorders (except for MA-induced sleep disorder), had no major axis psychiatric disorder unrelated to MA use (primary psychotic, mood, or anxiety disorders), had no intellectual disability, did not have the history of medical illnesses affecting sleep (e.g., metabolic diseases), and were in the early full remission phase. According to DSM-IV-TR criteria, the specifier of the early full remission phase is used when, “for at least 1 month, but less than 12 months, no criteria for dependence or abuse have been met”^20^. We excluded patients who relapsed on MA before completion of the study. To detect any relapses, we were directly asking the patients of any relapses and performing urine dipstick tests for methamphetamine and amphetamine. However, as all the participants were in the residential centers, no relapses were reported.

We asked all participants to respond to a researcher-made questionnaire for collecting demographic information. We have kept all the personal information confidential. To find out about the effect of abstinence on the sleep in former MA users, we perform PSG for all participants.

PSG is a gold standard test evaluating one’s sleep to find out probable sleep disorders. In order to perform PSG, a patient should sleep one night in a sleep laboratory. During the test, the electroencephalogram (EEG), the oxygen saturation, the electrocardiogram (EKG), the airflow at the nose and mouth, the snoring and its intensity, the electrooculogram (EOG), the chin, and leg electromyogram, and the chest and abdominal wall movements of the participant are monitored and recorded. To analyze PSG, a sleep specialist first extracts the sleep parameters from the recorded data. Then, the extracted parameters compared with the standard indexes of healthy individuals. The report summary usually contains the pattern of distribution of sleep stages (sleep architecture and sleep staging), sleep-related breathing problems, unusual limb movements, and unusual behaviors during sleep^21^.

As we aimed to evaluate the sleep architecture, we have reported the total sleep time (TST), total sleep period (TSP), sleep efficiency (SE), waking after sleep onset (WASO), total arousal index (TAI), spontaneous arousal index, sleep onset latency (SOL), REM sleep, overall non-REM sleep and its stages (N1, N2, and N3), REM pressure, and REM latency. TST is the amount of time a patient decided to sleep until getting up. TSP is the amount of time the patient actually sleeps. SOL is the duration of time between the patient’s attempts to sleep, and the time sleep is started. WASO is the amount of time of wakefulness occurring after sleep really starts. REM latency is the amount of time from the initiation of sleep to the first REM episode. All these parameters are described in minutes. TSP is divided into REM sleep and non-REM sleep stages. Each stage is calculated by dividing the duration of that stage to the duration of TSP. SE refers to the ratio of TSP/TST. These parameters are expressed in percentages^21^. The REM pressure is the number of REM sleeps one experiences through one night.

Before performing PSG, researchers were explaining the process for each participant in detail. Also, a technician was acquainting them of the terms and regulations of the sleep lab and the procedure, such as limitation of caffeinated drink during the day leading to PSG. In addition, participants had the right to visit the sleep lab environment on the day before. In the present study, PSG was conducted using Stellate Harmonie recorders software (Stellate Systems, Inc., Montréal, Canada). We asked the participants to go to bed at 9:30 p.m. Their brain activity (EEG) and eye movements (EOG) were monitored during the sleep. Sleep stages and parameters were scored and interpreted according to American Academy of Sleep Medicine Manual (AASM) for the Scoring of Sleep and Associated Events, version 2.2.

The collected data were analyzed by the Kolmogorov-Smirnov test, independent sample t-test, and chi-square test, using SPSS 16. The level of significance was considered at less than .05.

## RESULTS

A total of 12 participants voluntarily participated in the present study. All had the diagnosis of methamphetamine dependence disorder in the early full remission in a controlled environment (based on the DSM-IV-TR). Their sleep parameters were evaluated objectively by PSG. The participants aged 27-42 years with a mean age of 32.4±5.1 years. The preferred method of MA administration in all of them was smoking. Table 1 shows the demographic characteristics of the participants.

**Table 1:** Demographic variables of the participants of the study.

	Variable	Result
Mean age (year)	Male	32.4±5.1
Sex	Female	11
Mean weight (kg)		75.6±19.5
Mean height (cm)		177.3±11.1
Body mass index (kg/cm2)		25.4±4.8
Mean abstinence duration (day)		81.3±34.9

## SLEEP ARCHITECTURE

Table 2 summarizes the results of the sleep architecture parameters in the participants of the present study.

**Table 2:** Results of the sleep architecture parameters according to the polysomnography of the participants of the study.

Variables	Mean ± SD		t	df	p-value
	Participants	Healthy standards			
TSP (min)	403.0±52.9	-	-	-	-
TST (min)	333.6±79.1	360-480	-1.2	11	0.27
SE (%)	75.7±14.4	85-100	-2.2	11	0.047
WASO (min)	69.3±53.4	20-40	4.5	11	0.08
Total arousal index (number)	17.4±14.1	0-10	1.8	11	0.10
Spontaneous arousal index (number	606±5.6	0-10	-2.0	11	0.07
Overall Non-REM (%)	80.3±7.2	75-80	1.4	11	0.20
REM (%)	17.8±8.6	20-25	-1.9	11	0.08
N1 (%)	12.7±5.5	2-5	5.8	11	0.000
N2 (%)	44.5±9.5	45-55	-2.0	11	0.07
N3 (%)	23.3±11.3	5-15	4.1	11	0.002
REM latency (min)	116.2±68.5	15-20	5	11	0.000
REM pressure (number)	6.0±4.0	4-5	1.3	11	0.22

Notes: TST = Total sleep time; TSP = Total sleep period; SOL = Sleep onset latency; SE = Sleep efficiency; WASO = Wake after sleep onset; REM = Rapid eye movement; N = non-REM sleep stage.

The mean of TST and TSP of the participants were within normal ranges. However, we witnessed that the mean of TST was significantly lower than TSP in the participants (*p*=0.001). The participants’ mean SE was 75.7±14.4 percent. Considering the normal range of SE between 85-100%, our participants had significantly lower SE than the healthy population (*p*=0.047).

The sleep onset latency (SOL) of the participants was non-significantly higher than the healthy population (SOL=25.3±22.7, *p*=0.44). We tried to more clarify it in the result section, and highlighted the sentences related to the SOL measures.

The participants of the study experienced an increase in the WASO and the total arousal index (Table 2). However, the increase in none of them was statistically significant (*p*=0.08 and *p*=0.10, respectively). The spontaneous arousal index was in normal range (Table 2). We found out that the share of the REM sleep showed a non-significant decrease (*p*=0.08), while the share of overall non-REM sleep showed just a slight non-significant increase (*p*=0.20) compared to the healthy population. However, the N1 and N3 stages of sleep increased significantly in the participants of the study compared to the healthy population (*p*<0.001 and *p*=0.002, respectively).

Evaluation of REM sleep showed a considerable increase in the REM latency of participants compared to the healthy population (*p*<0.001) and a non-significant increase in the REM pressure (*p*=0.22).

## DISCUSSION

In this study, we decided to investigate whether sleep disturbances of patients with MA dependence may resolve after reaching full remission from MA. Therefore, we evaluated the sleep architecture of 12 participants with the diagnosis of MA dependence on the early full remission by PSG. Despite the considerable duration of abstinence, we witnessed some deviation in sleep parameters from normal sleep. Although, on average, patients were abstinent for more than 2 months, they still had decreased sleep efficiency, prolonged REM latency, and increased duration of stages 1 and 3 of non-REM sleep. Since we could not find any similar study on the sleep parameters of MA ex-users during the remission in the literature, we compared our results with surveys focused on earlier withdrawal states of MA.

Researchers have observed that many signs and symptoms of MA discontinuation syndrome alleviate in less than three weeks after drug cessation^22^. However, according to our findings, sleep disturbances in MA users are long-lasting side effects. Participants continued to experience sleep problems many weeks after they stopped using MA (mean abstinence duration was 81.3±34.9 days). This finding is consistent with the results of a descriptive study assessing the sleep quality of former MA users, which showed that more than 50% of the previous MA users had impaired sleep quality during the fifth week after MA discontinued^4^. In 2010, Brower and Perron^23^ hypothesized that protracted sleep problems could be a risk factor of relapse in any substance user. Although the relation between sleep disturbances and craving or relapse is unclear in psychostimulant consumers^23, 24^, and our knowledge is lacking on how the treatment of sleep problems affects the long-term outcome of these patients^25^, most researchers have suggested that the treatment of sleep problems would help prevent relapse^26, 27^. The high prevalence of sleep disturbances should be taken into account for the successful recovery of former MA users. Therefore, we highly recommend the assessment of sleep problems in every MA user and applying available treatments in patients with sleep disturbances.

We observed that although the sleep architecture during extended abstinence had similarities with the sleep architecture during MA abuse, it had differences with the sleep architecture of early abstinence. Researchers have shown that amphetamines consumers have reduced TST and REM sleep and increased SOL and REM latency^12, 28^. The initial effects of MA cessation, however, is sleep rebound characterized by an increased TST and REM sleep and decreased SOL and REM latency^11, 13, 14^. Gossop et al. (1982)^14^ found out that the sleep rebound lasts for almost one week and followed by a period of reduced sleep, which continues for about 2 weeks^14^. Nevertheless, we showed that the reduced sleep after MA cessation continues beyond 2 weeks. According to our study, patients had a non-significant reduced TST and REM sleep (*p*=0.27 and *p*=0.08, respectively), a non-significant increased SOL (*p*=0.44), and a significant increased REM latency (*p*<0.001) compared to the normal population. Failure to recover from the negative effects of MA on the sleep parameters during the remission suggests structural and functional damages in sleep-wake centers in MA ex-users. Koob et al. (ANO)^x^ have hypothesized a neurobiologic adaptation model in chronic substance users. At first, substances activate some processes directly by affecting their target brain circuits. Then opponent processes are activated to neutralize the initial effects of substances on the brain to maintain the brain homeostasis. As the opponent processes could remain activated after substance cessation, they are responsible for some withdrawal syndrome^11^. The neurobiologic adaptation of sleep-wake systems in chronic MA users could explain the sleep rebound in the early withdrawal states. However, MA has a great impact on the brain circuits. Alvarenga et al. (2011)^29^ suggested that stimulants can potentially be genotoxic in brain cells in animal models^29^. Amphetamines produce documented long-term monoaminergic neurodegeneration in humans^18^. As monoaminergic neurons play an essential role in sleep regulation^30^, prolonged damage in the sleep-wake homeostasis of chronic MA users is not unexpected. Possible damages within the sleep-wake system could explain the prolonged disturbances of sleep architecture after long-term abstinence.

SE is an important parameter in polysomnographic studies. SE is calculated by dividing TSP into TST^21^. Therefore, the difference between TST and TSP could have a negative influence on sleep efficiency. Our participants had the mean TST within normal range (333.6±79.1 minutes, respectively). However, they had a significantly lower TST than TSP (*p*=0.001). Therefore, they experienced mean sleep efficiency as low as 75.7±14.4%. Sleep efficiency has a strong association with subjective sleep quality. Sleep efficiency of 87% and more is needed for rating the quality of sleep as “good”^31^. So, the objective findings of PSG on the sleep quality are consistent with a descriptive study on quality of sleep in former methamphetamine users, where 52.2% of participants reported improper quality of sleep in the fifth week of abstinence^4^. Participants of our study showed an increase, yet not significant, in WASO and total arousal index (TAI), as well. In normal sleep, people usually experience less than 40 minutes of awakening and less than 10 arousals after their sleep initiates. We found out that patients in the abstinence period from MA had the mean WASO duration of almost 70 minutes and the mean TAI of 17 times (Table 2). Increasing in WASO is a good index of sleep continuity problem^32^, leading to a lower quality of sleep^31^. The higher arousal index is a reliable indicator of more fragmented sleep^33^, resulting in lower sleep quality, too.

Participants of the present study showed significant rises in N1 and N3 stages of sleep. In an experimental model evaluating the effect of a single dose of amphetamine, researchers reported an episode of increased N3 (slow-wave sleep) after amphetamine-induced wakefulness^34^. In some reports, early abstinence from stimulants was associated with increased N3, too^35^. Although they have reported an increased N3 in the early abstinence period from stimulants, we showed that this increase could be long lasting after discontinuation of MA. However, we witnessed an increase in N1, which was not reported in any similar studies. Recently, some researchers have suggested that PSG may not have enough specificity to define the N1 stage of sleep. They interpreted that the N1 stage defined by PSG is more a mixture of wakefulness before sleep initiation (which shows SOL) and the first stage of non-REM sleep (N1)^36^. Therefore, the increased N1 stage in our study may indicate a more prominent SOL (instead of a non-significant increased SOL we witnessed in the study) and a shorter real N1 stage, which is misdiagnosed by PSG.

In summary, the present study provides some evidence that noticeable sleep disturbances, including decreased sleep efficiency, increased REM latency, and increased duration of N1 and N3 stages remain in MA ex-users long after they reach full remission.

## LIMITATION

This study had some limitations. We did not access the patients’ sleep patterns before and during their use of MA. Most of the participants of the study were men, as a higher number of stimulant users are men in Iran. Also, for assessing the presence of MA withdrawal syndrome criteria, we only performed a psychiatric interview and did not use any standard questionnaire.

This study was the first study assessing the sleep architecture of methamphetamines users after prolonged abstinence by polysomnography. Also, the validity of the total abstinence was very high, as the participants were receiving treatment in residential centers.
